# Knowledge and attitudes about the use of emergency contraception among college students in Tamil Nadu, India

**DOI:** 10.1186/s42506-019-0030-9

**Published:** 2020-01-29

**Authors:** Prem Davis, Malaimala Sarasveni, Jayalakshmi Krishnan, Lekha Diwakara Bhat, Naveen Kumar Kodali

**Affiliations:** 1 Health Centre, Central University of Tamil Nadu, Thiruvarur, 610005 India; 2 Hospital, National Institute of Technology, Tiruchirappalli, India; 3 Department of Life Sciences, Central University of Tamil Nadu, Thiruvarur, India; 4 Department of Epidemiology & Public Health, Central University of Tamil Nadu, Thiruvarur, India

**Keywords:** Emergency contraception, College students, Abortions, Unwanted pregnancy

## Abstract

**Background:**

In India, a large number of pregnancies are unplanned resulting in unsafe and illegal abortion. For every legal abortion, 10 to 11 illegal abortions are occurring which endangers the health and survival of the women. In recent years, there is an increase in unwanted and unintended pregnancy at the early age group. Usage of emergency contraception (EC) can decrease the unwanted pregnancy and provide a healthier life.

**Aim:**

The aim of study is to assess the knowledge and attitude regarding EC among college students in Thiruvarur District, Tamil Nadu, India.

**Methods:**

A cross-sectional study was conducted among the college students of Thiruvarur district, Tamil Nadu, India. A total of 758 students were selected by convenient sampling technique. Data was collected by administering a pretested semi-structured questionnaire.

**Results:**

Out of 758 students, 183(24%) heard about EC. The commonest source of information was the internet 91 (49.7%). The majority 116 (63.4%) knew that it does not prevent STDs. Of those who were aware, 42% were aware of 42.6% are aware of the time limit to use EC. The knowledge level of about the EC was moderate (60.1%), and it was significantly (*p* < 0.05) more among students > 25 years old, married participants, students in private institution, of lower socio-economic status, Muslim students and days’ scholars. The negative attitude towards EC was 59%. Nearly 38.8% believed that the EC will affect the next menstrual period, and 35.5% informed it will increase high risk behaviour among adults. The attitude level was significantly associated with the same factors associated with the awareness level with the factors Christian religion replacing Muslim and higher socioeconomic class replacing lower class participants.

**Conclusion:**

The knowledge level of the studied college students was moderate, and they mostly had negative attitude towards the EC. Reproductive health education should be given in educational institutions to promote awareness and to remove misconceptions about EC.

## Introduction

In today’s time, the population growth has become one of the leading problems in the world. According to 2019 data, nearly about 7.7 billion people are living in the world [1]. India contributes to 1210 million population, and every year, it is adding 17.5 million people newly. At present, this overpopulation is the major problem in the country, which leads to problems like poverty, illiteracy, decrease in the economic growth, starvation, malnutrition, depletion of natural resources and unemployment [2].

Worldwide, it is estimated that 44% of the pregnancy occurring were unintended between 2010 and 2014 [3]. In the same interval, 55.7 million abortions occurred worldwide, of which 25.1 million abortions were unsafe abortions, mostly happening in the developing countries [4]. In India, 78% of the pregnancies are unplanned. And it is estimated that nearly 25% of them are unwanted. Every year nearly 11 million abortions take place of which 6.7 million are induced and 4 million are spontaneous abortions. Under the preview of MTP act, abortions are permitted in India since 1971 for specific indications. However, approximately 10 to 11 illegal abortions for each legal abortion are occurring. Nearly 20,000 women are dying annually due to abortion-related complications which are almost preventable [5]. Thus, unwanted and unintended pregnancies play a major role in the reproductive health of young adults.

The emergency contraception (EC) is a contraceptive method used to prevent unwanted pregnancy in the first few days after the unprotected sexual intercourse or contraceptive failure/accident. The EC is also known as post-coital pills or morning-after pills [5]. According to WHO, EC can prevent up to 95% of the pregnancies [6]. In many developed countries, there is an increased incidence of high-risk sexual behaviour among the adolescents’ age group, and their awareness level of the EC were excellent with the percentage varying from 61 to 93% [7–9]. Surprisingly, in developing countries also there is an increased trend of sexual activity at an early age. By the age of 18 years, 40–80% of females become sexually active [10]. But their awareness level regarding the EC is low compared with the developed countries. Limited access to information and services often cause major reproductive health problems to young women due to unwanted pregnancy or unsafe/illegal abortions [11]. India is also facing similar problems like other counties. Though Tamil Nadu is one of the best states in providing health care service in India [12], only one previous study was conducted among college students and it was in private colleges in the state. This study was planned to assess the awareness and attitude of college students in Tamil Nadu regarding EC.

## Materials and methods

This cross-sectional study was conducted among the college-going students of Thiruvarur District in Tamil Nadu from February 2019 to April 2019.

### Sampling

Considering the awareness regarding EC among college students is 23.1% [13]. Taking alpha error as 5% and absolute margin of error as 3% [13], the sample size was calculated to be 758. All the colleges of the district (*n* = 15) were contacted and informed about the nature of the study. Due to its sensitiveness, finally one government university and two private colleges agreed to participate in the study after assuring that the name of colleges will not be disclosed at any point in time.

### Data collection

The questionnaire was pretested on 5% of the students prior to the actual data collection of the study population. This was done to assess the ambiguity and comprehensibility of the questionnaire and subsequent modification was done for the ease of comprehension. The students who are included in the pretesting were excluded from the study. The content validation of the questionnaire was done by experts in the research subject from the departmental research committee of the Institute. The participants were chosen by convenient sampling methods. All the students of the colleges were approached and were informed about the objectives of the study and assured that the information collected will be kept confidential. The students who gave written consent were given a pre-tested (modified after pretesting) self-administered anonymous questionnaire with a condition that all the questions to be answered compulsorily. The students actively get the questionnaire and enthusiastically gave their response with a net response rate 91%. The anonymity of the participants was also assured and ensured.

### Variables of the study

The semi-structured questionnaire consisted of three parts. The first part consisted of the basic demographic details like age, gender, marital status, course, year, institution type, place of matriculation and socio-economic status based on modified BG Prasad scale [14], religion and residence. The second part consists of 17 knowledge questions with multiple choices regarding EC, and some questions have multiple responses. Each correct answer was scored “1”. Based on the total scores, the level of awareness was divided into poor, moderate and high awareness. The cumulative score below 8 was considered as poor awareness, the score of 9 to 17 was considered as moderate awareness and score of 18–25 was considered as high awareness. The third part consists of the attitude of the participants regarding EC. It consists of nine statements with responses of “Agree”, “Neutral”, “Disagree” These responses were scored on a three-point Likert scale: a score of “2” was assigned for a favourable response, “1” for neutral response and “0” for the unfavourable/negative response. Scores ranged from 0 to 18 and the cumulative score ranging between 0 and 12 was considered as negative attitude, and score of 13 to 18 was considered as positive attitude towards the EC.

### Statistical analysis

All the data were entered in Excel and analysed through SPSS version 20 software. Chi-square test was used to test the association. And the *p* value of < 0.05 was considered significant.

## Results

A total of 758 students participated in the study and filled the questionnaire. The mean age of the participants was 22.9 ± 4.3 years. The majority were females 419 (55.3%), less than 25 years old 493 (65%), unmarried 649 (85.6%) and belonging to upper socio-economic status 331 (43.7%). The study was conducted among the students of various disciplines/colleges pursuing undergraduate, post graduate and doctorate degrees. And for better understanding, all the courses were grouped in 4 main branches based on its nature. The branches include the background of arts, science, professional courses and research scholars. The majority of the study participants are the students of arts course 299 (39.4%), second year of their course 372 (49.1%), from the government institutions 409 (54%) and 402 (53%) did schooling in urban areas; 526 (69.4%) were Hindu by religion and 426 (52.6%) were hostellers as shown in Table 1

**Table 1:** Socio-demographic characteristics of the college student’s sample in Thiruvarur District, Tamil Nadu, India (*n* = 758)

Characteristics	*N*	%
Age		
Less than 25	493	65
25 and above	265	35
Gender		
Female	419	55.3
Male	339	44.7
Marital status		
Married	101	14.4
Unmarried	649	85.6
Course^#^		
Arts	299	39.4
Science	235	31.0
Professional	170	22.4
Research scholars	55	7.2
Year		
I	303	40.0
II	372	49.1
III	83	10.9
Institution type		
Government	409	54.0
Private	349	46.0
Place of matriculation		
Rural school	356	47.0
Urban school	402	53.0
Socioeconomic status (modified BG Prasad scale)		
Rs 6574 and above	331	43.7
Rs 3287 to 6573	99	13.1
Rs 1972 to 3286	148	19.5
Rs 986 to 1971	125	16.5
Rs 985 and below	55	7.2
Religion		
Christian	130	17.2
Hindu	526	69.4
Muslim	102	13.4
Residence		
Day scholar	332	43.8
Hosteler	426	56.2

^#^Professional courses: Students who are pursing undergraduate and postgraduate degree in management and engineering were grouped in professional courses. Students who are pursuing PhD in any branch are under research scholar category

**Fig. 1:**
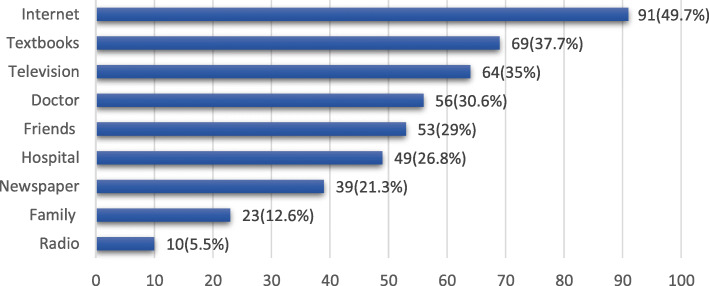
The source of information about the emergency contraception (multiple response question)

Out of 758 participants, 183 (24.1%) had heard about the EC. Among them, males comprised 39.9% and females comprised 60.1%. The main source of information about EC among participants was internet 49.7%, textbooks 37.7%, television 35%, doctors 30.6%, friends 29%, hospital 26.8, newspaper 21.3%, family 12.6% and radio 5.5% as shown in Fig. 1.

Out of 183 (24.1%) who had knowledge about the EC, 42.1% had knowledge that combined oral contraceptive pills (OCPs) can be used as an EC followed by 23% progesterone only pills. About 31.7% of the participants were aware that intra uterine contraceptive device (IUCD) can be used as EC, and 20.2% were aware about the maximum time limit to IUCD as EC as within 5 days.

Most of the participants have knowledge regarding the brand of ECPs, 31.1% opted for I-pill followed by 27.9% for Mala-N, 12.6% Ezy pill and 7.1% for Ovral while 37.7% were unaware about this. Common places preferred by the participants to purchase EC were local pharmacy shops 64.5%, government hospitals 61.7% followed by private hospitals 35.5%.

A considerable proportion of the participants who were aware of EC had knowledge regarding the indications of EC, 56.8% after unprotected sex, 50.8% as a birth control measure, 48.1% following failure of contraception, 42.6% following forced sex/sexual assault and 42.1% following rape. Only 8.2% of the participants lack knowledge about the indication.

The majority of the participants 63.4% were aware that the EC will not protect against HIV/AIDS and other sexually transmitted diseases. 66.2% of the participants reported that consultation is necessary before taking EC. Nearly half 48.1% of the participants reported that pregnancy test is required before taking EC.

About 42.6% opted that the OCPs can be taken within 72 h of the unprotected sex followed by 13.7% within 24 hours. The majority of the participants 77.6% were aware of the failure rate/effectiveness of the OCPs that failure can happen in spite of taking EC, and only a few (12, 6.6%) had a misconception that it is always effective.

Most of the study participants 63.4% were unaware of the exact recommended doses of OCPs, and 63.9% were unaware of the recommended time interval between the doses of OCPs as shown in Table 2.

**Table 2:** Knowledge regarding the EC among the college students who had heard about the EC (*n* = 183)

Characteristics	*N*	%
Medications used as EC		
OCPs	77	42.1
Progesterone only pills	42	23.0
Antibiotics like amoxicillin, erythromycin	3	1.6
Do not know	61	33.3
Trade names^#^		
I Pill	57	31.1
Ezy Pill	23	12.6
Mala-N	51	27.9
Ovral	13	7.1
Do not know	69	37.7
Place of availability^#^		
Government hospital	113	61.7
Private hospital	65	35.5
Pharmacy	118	64.5
Supermarket	8	4.4
Any shops	5	2.7
Do not know	9	4.9
Price (INR)		
Less than 100	61	33.3
100 to 200	37	20.2
200 to 300	3	1.6
Do not know	82	44.8
Indications for EC^#^		
Unprotected sex	104	56.8
Forced sex/sexual assault	78	42.6
Rape	77	42.1
Birth control measure	93	50.8
Failure of contraception	88	48.1
To induce abortion	33	18
Do not know	15	8.2
Requirement of doctor consultation before taking EC		
Yes	121	66.2
No	31	16.9
Do not know	31	16.9
EC prevent STD		
Yes	43	23.5
No	116	63.4
Do not know	24	13.1
Requirement of pregnancy test before taking EC		
Yes	88	48.1
No	44	24
Do not know	51	27.9
Time at which OCPs to be taken		
Within 24 h after sex	25	13.7
Within 72 h after sex	78	42.6
Within 5 days after sex	7	3.8
Before sex	14	7.7
Do not know	59	32.2
Effectiveness in pregnant women		
Yes	23	12.6
No	115	62.8
Do not know	45	24.6
Effectiveness in preventing pregnancy		
Always effective	12	6.6
Failures can happen	142	77.6
Do not know	29	15.8
EC are same as abortion pills		
Yes	17	9.3
No	125	68.3
Do not know	41	22.4
Recommended number of doses		
One dose	26	14.2
Two doses	39	21.3
Three doses	2	1.1
Do not know	116	63.4
Recommended time between the doses		
12 h apart	9	4.9
24 h apart	21	11.5
48 h apart	34	18.6
To be taken with the pill	2	1.1
Do not know	117	63.9
Frequency in a month		
Once	36	19.7
Twice	11	6
Thrice	4	2.2
Daily	4	2.2
weekly	4	2.2
Do not know	124	67.7
Can IUCD used for EC		
Yes	58	31.7
No	39	21.3
Do not know	86	47
Timing of insertion of IUCDs		
Within 5 days	37	20.2
Within 7 days	4	2.2
Within 1 month	1	0.5
At the time of sexual intercourse	18	9.8
Do not know	123	67.2

^#^Multiple responses

The majority of the participants were aware about the side effects of EC; the common side effects of EC as stated by the participants were menstrual irregularities 60.7%, abdominal pain 40.4%, vomiting 38.3% and nausea 31.1%, fever 11.5% while 30.6% of the participants were unaware of the side effects of the EC.

**Fig. 2:**
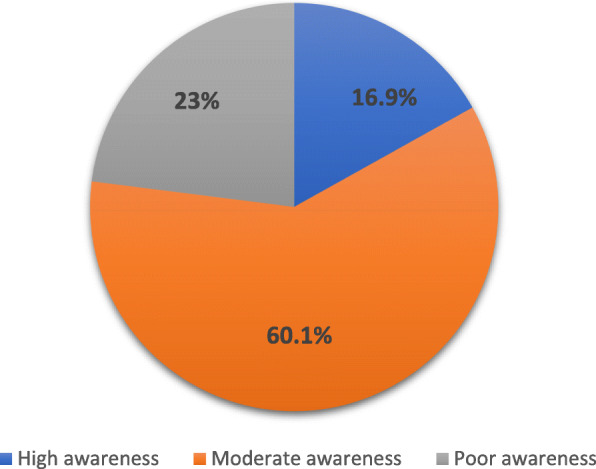
Distribution of the college students according to the level of awareness (*n* = 183)

The awareness levels of the students were poor awareness 23%, moderate awareness 60.1% and high awareness 16.9% as shown in Fig. 2

On assessing the association between the sociodemographic variables with the awareness level of the study participants, there is a strong association seen with the participants of more than or equal to 25 years of age (*p* < 0.001), married participants (*p* = 0.027), private college students (*p* = 0.002), class IV socioeconomic status (*p* < 0.001), Muslim community participants (*p* = 0.001) and days' scholars (*p* = 0.001). However, variables like gender (*p* = 0.946), course (*p* = 0.080), year of the study (*p* = 0.704), and place of matriculation (*p* = 0.459) were not associated with the awareness level about the EC among the participants as shown in Table 3.

**Table 3:** Association between the socio-demographic variables and the awareness level of college students regarding the emergency contraception (*n* = 183**)**

Characteristics	Awareness level				*X*^2^ (*p* value)
	Poor (%) (1–8)	Moderate (%) (9–17)	High (%) (18–25)	Total *N* (%)	
Age					
Less than 25	36 (29.8)	72 (59.5)	13 (10.7)	121 (100)	15.3
25 and above	6 (9.7)	38 (61.3)	18 (29)	62 (100)	(< 0.001)
Gender					
Female	26 (23.6)	66 (60)	18 (16.4)	110 (100)	0.1 (0.946)
Male	16 (21.9)	44 (60.3)	13 (17.8)	73 (100)	
Marital status					
Married	4 (10.3)	24 (61.5)	11 (28.2)	39 (100)	7.2 (0.027)
Unmarried	38 (26.4)	86 (59.7)	20 (13.9)	144 (100)	
Course					
Arts	18 (31.6)	27 (47.4)	12 (21.1)	57 (100)	
Science	15 (21.1)	50 (70.4)	6 (8.5)	71 (100)	11.3 (0.080)
Professional	4 (14.3)	18 (64.3)	6 (21.4)	28 (100)	
Research Scholars	5 (18.5)	15 (55.6)	7 (25.9)	27 (100)	
Year					
I	15 (26.8)	33 (58.9)	8 (14.3)	56 (100)	
II	20 (19.6)	62 (60.8)	20 (19.6)	102 (100)	2.2 (0.704)
III	7 (28)	15 (60)	3 (12)	25 (100)	
Institution type					
Government	33 (29.2)	68 (60.2)	12 (10.6)	113 (100)	11.9 (0.002)
Private	9 (12.9)	42 (60)	19 (27.1)	70 (100)	
Place of matriculation					
Rural school	17 (20.5)	49 (59)	17 (20.5)	83 (100)	1.5 (0.459)
Urban school	25 (25)	61 (61)	14 (14)	100 (100)	
Socioeconomic status					
Rs 6574 and above	33 (29.5)	71 (63.4)	8 (7.1)	112 (100)	
Rs 3287 to 6573	1 (4)	13 (52)	11 (44)	25 (100)	34.7
Rs 1972 to 3286	4 (14.3)	18 (64.3)	6 (21.4)	28 (100)	(<0.001)
Rs 986 to 1971	2 (15.4)	8 (61.5)	3 (23.1)	13 (100)	
Rs 985 and below	2 (40)	0 (0)	3 (60)	5 (100)	
Religion					
Christian	4 (12.1)	23 (69.7)	6 (18.2)	33 (100)	
Hindu	34 (26.6)	79 (61.7)	15 (11.7)	128 (100)	17.9 (0.001)
Muslim	4 (18.2)	8 (36.4)	10 (45.5)	22 (100)	
Residence					
Day scholar	8 (13.8)	32 (55.2)	18 (31)	58 (100)	
Hosteler	34 (27.2)	78 (62.4)	13 (10.4)	125 (100)	13.4 (0.001)

*p* < 0.05 is considered significant

Out of 183 participants, 55.2% disagreed that the EC is promoting promiscuity (immoral). Only 29% of the participants had misconception that EC is a method of abortion. Most of them 71% disagreed for EC as a sinful act. Nearly 38.8% were neutral for the question, whether EC may affect the baby and half of the participants disagreed that the use of EC leads to infertility. Some 38.8% of the study participants agreed that the usage of EC will affect the next menstrual period. Most of the participants 60.1% agreed that they will advise EC to others and 35.5% agreed that the use of EC will increase the high-risk behaviour among the youths. The majority 82.5% gave positive response that the knowledge of EC should be given in the educational institutions as shown in Table 4.

**Table 4:** Attitude of college students towards the emergency contraception (*n*-183)

Characteristics	Attitude		
	Agree (%)	Neutral (%)	Disagree (%)
EC promoting promiscuity (immoral)	23 (12.6)	59 (32.2)	101 (55.2)
EC is a method for inducing abortion	53 (29)	32 (17.5)	98 (53.6)
EC a sinful act	18 (9.8)	35 (19.1)	130 (71)
EC use will lead to infertility	34 (18.6)	58 (31.7)	91 (49.7)
EC may affect the baby if it does not work	48 (26.3)	71 (38.8)	64 (35)
EC will affect the next menstrual period	71 (38.8)	61 (33.3)	51 (27.9)
Will you advice EC for others	110 (60.1)	41 (22.4)	32 (17.5)
The use of EC will encourage high risk behaviour among youths	65 (35.5)	59 (32.2)	59 (32.2)
The information and knowledge regarding the EC have to be given in educational institutions	151 (82.5)	25 (13.7)	7 (3.8)

**Table 5:** Association between sociodemographic variable and attitude of college students regarding emergency contraception (*n* = 183)

Characteristics	Attitude level			*X*^2^ (*p* value)
	Negative attitude (0–12)	Positive attitude (13–18)	Total *N* (%)	
Age				
Less than 25	86 (71.1)	35 (28.9)	121 (100)	21.5 (< 0.001)
25 and above	22 (35.5)	40 (64.5)	62 (100)	
Gender				
Female	69 (62.7)	41 (37.3)	110 (100)	1.6 (0.210)
Male	39 (53.4)	34 (46.6)	73 (100)	
Marital status				
Married	16 (41)	23 (59)	39 (100)	6.6 (0.010)
Unmarried	92 (63.9)	52 (36.1)	144 (100)	
Course				
Arts	44 (77.2)	13 (22.8)	57 (100)	24.1 (< 0.001)
Science	45 (63.4)	26 (36.6)	71 (100)	
Professional	7 (25)	21 (75)	28 (100)	
Research scholars	12 (44.4)	15 (55.6)	27 (100)	
Year				2.5 (0.286)
I	37 (66.1)	19 (33.9)	56 (100)	
II	55 (53.9)	47 (46.1)	102 (100)	
III	16 (64)	9 (36)	25 (100)	
Institution type				22.4 (< 0.001)
Government	82 (72.6)	31 (27.4)	113 (100)	
Private	26 (37.1)	44 (62.9)	70 (100)	
Place of matriculation				0.8 (0.362)
Rural school	52 (62.7)	31 (37.3)	83 (100)	
Urban school	56 (56)	44 (44)	100 (100)	
Socioeconomic status				13.1 (0.011)
Rs 6574 and above	76 (67.9)	36 (32.1)	112 (100)	
Rs 3287 to 6573	12 (48)	13 (52)	25 (100)	
Rs 1972 to 3286	12 (42.9)	16 (57.1)	28 (100)	
Rs 986 to 1971	4 (30.8)	9 (69.2)	13 (100)	
Rs 985 and below	4 (80)	1 (20)	5 (100)	
Religion				9.4 (0.009)
Christian	12 (36.4)	21 (63.6)	33(100)	
Hindu	80 (62.5)	48 (37.5)	128(100)	
Muslim	16 (72.7)	6 (27.3)	22(100)	
Residence				24.2 (< 0.001)
Day scholar	19 (32.8)	39 (67.2)	58(100)	
Hosteler	89 (71.2)	36 (28.8)	125(100)	

*p* < 0.05 is taken as significant

**Fig. 3:**
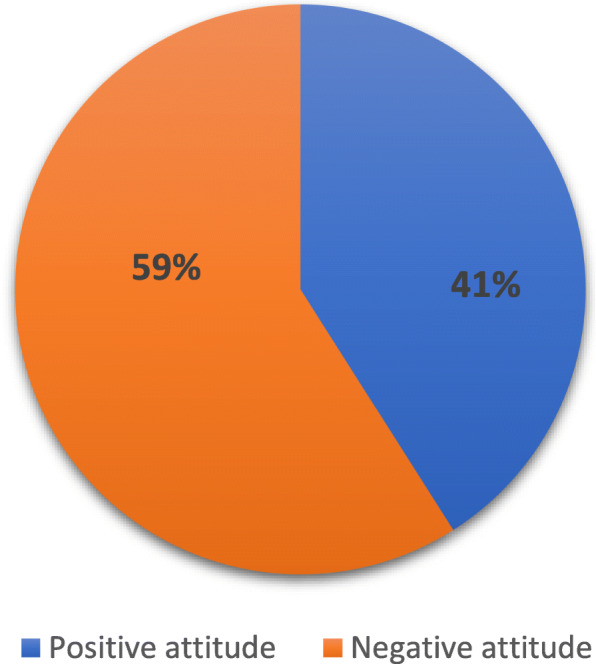
Distribution of college students according to the attitude levels toward emergency contraception (*n* = 183)

Out of 183 participants who heard of EC, nearly 59% had negative attitude towards the use of EC as shown in Fig. 3.

On assessing the association between the socio-demographic variables with the attitude levels of the participants, significant associations were seen with the participants who are 25 years and above (*p* < 0.001), married participants (*p* = 0.010), professional group (*p* < 0.001), from private institutions (*p* < 0.001), class IV socioeconomic status (*p* = 0.011), Christian community participants (*p* = 0.009) and hostellers (*p* < 0.001). However, variables like gender (*p* = 0.210), year of the study (*p* = 0.286) and place of matriculation (*p* = 0.362) were not associated with the attitude level about the EC among the study participants as shown in Table 5.

With regards to association between the knowledge score and the attitude score of the participants towards the EC, there is a significant association noted, the negative attitude is associated with poor and moderate awareness regarding the EC (*p* < 0.001) (Table 6)

**Table 6:** Association between the knowledge score and attitude score towards emergency contraception among college students (*n* = 183)

Characteristics		Awareness level			Total	*X*^2^ (*p* value)
		Poor awareness (%)	Moderate awareness (%)	High awareness (%)		
Attitude level	Negative attitude	36 (33.3)	62 (57.4)	10 (9.3)	108 (100)	21.9 (< 0.001)
	Positive attitude	6 (8)	48 (64)	21 (28)	75 (100)	
Total		42	110	31	183	

## Discussion

This study aimed to assess the knowledge and attitude on EC among the college students and to find the association between the socio-demographic variables with the knowledge and attitude score. Only 24.1% of the participants heard about the EC which is similar to another study conducted in college students in Puducherry which showed that 23.1% were aware of the EC [13]. More females 60.1% heard about EC when compared with males 39.9%. The reason could be that the wide availability of information about EC in the internet, newspaper, radio etc., and also the EC methods are widely used by females. Similar to this study, a study conducted in the USA showed significantly greater proportion of the female students had heard about the EC than the males [15].

Nearly half of the study participants opted for the internet as the common source of information which might be due to the easy accessibility of the high-speed internet through the smartphone’s in the recent times followed by textbook which shows most of the students had a knowledge about the EC in their school days. A similar study at Mangalore, India, showed that television was the most common source of information [16]. In this study, the majority of the participants had correct knowledge that combined oral contraceptive pills can be used as EC. A similar study conducted at Ethiopia by Grima et al. also showed that oral contraceptive pills can be used as EC [17]. This shows that most of the students know the medications to be used as EC. In our study, 31.7% of the students knew that the IUCD can also be used as EC and 20% of them knew that it should be inserted within 5 days of unprotected sexual intercourse. This shows that the study participants have knowledge regarding the IUCD, their use as EC and their maximum time limit of insertion to prevent pregnancy. A study conducted by Tilahun et al. in Ethiopia also showed that IUCD can be used as an EC, and in the same study, only 3.5% of the study participants reported that the IUCD should be inserted within 5 days of unprotected sexual intercourse [18].

I-pill was the most commonly known brand of OCPs among students of the present study. Similarly, the study at Mangalore also shows that the same brand is known among the study participants [16]. Nearly 64.5% of the students informed that EC can be purchased from local pharmacy shops followed by government (61.7%) and private (35.5%) hospitals. A study conducted in female university students at South Africa also showed that the public health facilities are the common source of getting EC [19].

The unprotected sex, birth control measure and following the failure of the contraception were participants’ responses for the indication of the EC, indicating that the participants were aware of the situations in which EC should be used. This may be due to the widespread knowledge of EC among students. A significant number of the students (63.4%) reported that it will not prevent the sexually transmitted diseases, and almost half of them were aware to have a pregnancy test before taking EC. But the study conducted at Gujarat, India, among undergraduate medical students showed that nearly 75% of the participants reported that the EC will prevent sexually transmitted diseases, and a significant number of the students mentioned that there is no need to do pregnancy test before taking EC [20].

About 42.6% of the study participants opted that the OCPs can be taken up to 72 h followed by 13.7% opted for within 24 h after the unprotected sexual intercourse. A similar study conducted by Shiferaw et al. among the female university students in Ethiopia showed that 36.1% of the students, opted that it should be taken within 24 h followed by 28.3% reporting the timing as within 72 h after the unprotected sex [21]. In our study, a significant number of participants opted that failure can happen after taking an EC, and they were aware that it is not the same as that of the abortion pills. However, the study did not investigate the failure rate of each EC among the participants. A similar study conducted at Gujarat, India also showed that a significant number of the students knew that the EC, and the abortion pills are different [20].

In our study, about 60% opted for the menstrual irrigularity as the most commom side effect followed by abdominal pain (40.4%), vomiting (38.3%) and nausea (31.1%). A similar study conducted at Ahmedabad, India, by Shelat et al. [22] among college students showed the most common side effect is nausea and vomiting followed by menstrual irregularities.

In our study on assessing the association between the sociodemographic variables with the awareness level, there is a significant association seen with participants > 25 years of age, married participants, private college students, class IV and class V socioeconomic status, Muslim community participants and days’ scholars. However, other variables like gender, course, year of the study and place of matriculation did not show any association. Similarly, a study by Tilahun et al. in Ethiopia also showed significant association with the age, course of the study and knowledge level [18]. The Ethiopian study by Nibabe et al. showed a significant association with the marital status and knowledge level [23]. A study in Nigeria by Awolke et al. showed a significant association between the socio-economic status and the knowledge level [24]. A study at Tigray, Ethiopia by Gebrehiwot et al. [25] showed association with the private college students with the knowledge level. A study at Lucknow, India, by Mishra et al. [26] showed significant association with the religion, and the study by Hoque et al. [19] in South Africa showed significant association with the day’s scholar with the knowledge level similar to our study. The Mangalore study by Joseph et al. [16] showed a significant association with the gender which is against our study results and no association with the place of matriculation and the knowledge level which is similar to our study. The studies in Mangalore and Turkey showed an association between the course and the knowledge level which is not seen in our study [16, 27]. Similar to our study, a Nigerian study by Arinze-Oniya et al. also showed no association with the year of the study and the knowledge level [28].

Regarding the attitude about the EC, 55.2% disagreed that the EC promots promiscuity. A similar study by Shiferaw et al. in South West Ethiopia also showed that EC is not promoting promiscuity [21]. But the Mangalore [16] and Raipur study [29] showed a neutral response towards promiscuity associated with the use of EC. Only 29% of our participants agreed that EC is a method of abortion. But the Ethiopian study by Shiferaw et al. showed half of the participants did not know whether the EC leads to abortion or not [21]. Meanwhile, the majority (71%) disagreed that EC is a sinful act and does not lead to infertility in a woman. In contrary, participants in the study by Shiferaw et al. showed that the EC will lead to infertility [21]. A large percentage of the participants 38.8% believed that the use of EC will affect the next menstrual period. Similar to this, Shiferaw et al. also showed the EC will affect the next menstrual period [21]. But the study by Tajure et al. shows the majority of the participants (50.2%) mentioned that it will not have any effect on next menstrual period [30]. Sixty percent will recommend EC to others which is similar to studies conducted at Ahmadabad [22] and Mangalore [16]. In our study, only 35.5% of the participants believed that EC will increase high-risk behaviour among youths. Other studies in Nigeria [31] and Trinidad [32] also showed similar results. Most of the students (82.5%) agreed to have the knowledge of EC in educational institutions. Similarly, the South African study by Hoque et al. also showed that 70.3% of participants agreed to have the knowledge of EC in study programmes [19].

On assessing the attitude level, there is significant association seen with the participants who are 25 years and above, married participants, professional group, private institutions, class IV socioeconomic status, Christian participants and days’ scholar. A study in Adama, Ethiopia, also showed a significant association between the age of the participants, marital status and religion with the attitude level [18]. Another study at Uttar Pradesh, India, also showed association with the course of study and religion with the awareness level of the participants [26]. In our study, the participants with a positive attitude are significantly and most likely to have moderate awareness regarding EC. Studies by Gajera et al. [20] and Bugssa et al. [33] also showed significant association between the knowledge and attitude level of the participants.

### Limitations of the study

As convenience sampling was used in the study, so the sample may not represent the target population. Though the anonymity was ensured, the accuracy of the response provided by participants cannot be guaranteed since the EC is a sensitive issue.

## Conclusions

The accurate knowledge about EC was low. The majority of the participants had moderate awareness and a negative attitude about it. So, there is a need to improve the knowledge and the attitude level of the participants about EC. Strategies to promote EC should be developed through IEC materials, and information about EC should be given within the curricula of the educational institutions. Many awareness programs should be conducted regarding EC among college-going students. For the prevention of the unwanted, unintended pregnancy and abortion, EC should be promoted along with the family planning methods in the health care settings. If these factors are addressed through appropriate health education methods, the morbidity and the economic burden could be averted.

## Data Availability

The datasets used and/or analysed during the current study are available from the corresponding author on reasonable request.
